# Modified and alternative Baveno VI criteria based on age for ruling out high-risk varices in patients with compensated cirrhosis

**DOI:** 10.1007/s12072-022-10359-y

**Published:** 2022-06-21

**Authors:** Lili Zhao, Ting Wang, Chunxia Guo, Li Zhou, Ping Han, Chunyan Wang, Ying Ma, Jing Wang, Min Gao, Jia Li

**Affiliations:** 1 Department of Gastroenterology and Hepatology, Tianjin Second People’s Hospital, Tianjin, China; 2 Department of Gastroenterology and Hepatology, Second People’s Clinical College of Tianjin Medical University, Tianjin Second People’s Hospital, Tianjin, China

**Keywords:** Liver cirrhosis, High-risk varices, Age, Ultrasound, Liver stiffness

## Abstract

**Background:**

The Baveno VI criteria (B6C) have been recommended to screen high-risk varices (HRV) in patients with liver cirrhosis to avoid the use of esophagogastroduodenoscopy (EGD). Due to conservative nature of B6C and the general unavailability of transient elastography in the medical institutions, clinical application of B6C is restricted. We aimed to optimize B6C and attempted to replace the liver stiffness (LS) score with other parameters that could help patients avoid EGD.

**Methods:**

A total of 1,188 patients with compensated cirrhosis were analyzed and divided into the training cohort (TC) and validating cohort (VC) by the split-sample method. Variables were selected to develop new criteria in the TC before verification in the VC.

**Results:**

The parameters of age ≥ 50 years, LS, platelet count (PLT), and spleen area (SA) were independently associated with HRV. The risk of HRV was 2.39 times greater in patients over 50 years, hence alternative B6C (AB6C) and modified B6C (MB6C) criteria were built based on age. MB6C was built by adjusting the cut-off value of LS and PLT (patients aged < 50 years with PLT > 100 × 10^9^/L and LS < 30 kPa; patients aged ≥ 50 years with a combined PLT > 125 × 10^9^/L and LS < 20 kPa). MB6C helped avoid EGD in 310 (51.2%) patients, whereas 7 (2.3%) cases of HRV were missed. The predicting performance HRV showed no statistical difference between PLT, SA, or LS. SA was selected to replace LS and in the built AB6C (patients aged < 50 years with PLT > 100 × 10^9^/L and SA < 55 cm^2^; patients aged ≥ 50 years with a combined PLT > 125 × 10^9^/L and SA < 44 cm^2^). Using AB6C avoided 297 (49.1%) EGDs with a total of 8 (2.7%) cases of HRV that were missed.

**Conclusions:**

Our novel MB6C and AB6C were stratified by age and provided excellent performance for ruling out HRV, which performed better than B6C and EB6C (expanded B6C) in helping to avoid EGD screening.

**Clinical trial registration number:**

ChiCTR-DDD-17013845.

**Supplementary Information:**

The online version contains supplementary material available at 10.1007/s12072-022-10359-y.

## Introduction

Gastroesophageal variceal hemorrhage is one of the most serious decompensation events that directly affects patient survival rate [1]. Within the last few years, international guidelines [2] arrived at the consensus that “all cirrhotic patients should be screened for varices at diagnosis.” However, the reported prevalence of high-risk varices (HRV) is as low as 16% in patients with well-compensated cirrhosis [3]. Considering the invasiveness and low acceptance of esophagogastroduodenoscopy (EGD) in patients, it is necessary to develop non-invasive methods to evaluate the presence of varices. The 2015 Baveno VI Conference consensus recommended that platelet count (PLT) and liver stiffness (LS) be assessed to identify patients with HRV (Baveno VI criteria, B6C) [4]. Patients with compensated liver cirrhosis (CLC) who meet the criteria (LS < 20 kPa and PLT > 150 × 10^9^/L) avoid undergoing EGD assessment as risk of developing HRV is considered acceptably low (risk < 5%). Although B6C has been shown to be effective in preventing 16–33% of EGDs [5–7], it remains perceived as conservative.

The expanded Baveno VI criteria (EB6C) (PLT < 110 × 10^9^/L and LS > 25 kPa) helped to avoid up to 40% of EGDs [8]. However, among the validations of B6C and EB6C, inconsistent conclusions were drawn owing to the etiological differences of cohorts [9–11] and a limited Asian cohort with hepatitis B. Therefore, verification and optimization of B6C are needed in large-scale cohorts. Additionally, a substitution for LS is required as transient elastography (TE) is not widely accessible in all medical institutions. Meanwhile, a growing number of early cirrhosis diagnoses have been confirmed by the progressive development of non-invasive methods. In light of these reasons, it is necessary to optimize the B6C to rule-out more patients from undergoing EGD.

In this study, we aimed to identify the risk factors that contribute to HRV and to optimize B6C (modified Baveno VI criteria, MB6C). Consequently, “easy-to-use” criteria (alternative Baveno VI criteria, AB6C) with more accuracy were developed using other laboratory variables as alternatives to LS. Furthermore, we directly compared the performance of MB6C and AB6C with B6C and EB6C.

## Patients and methods

### Patient selection

Demographic, laboratory, and clinical data were collected for all patients. Clinical data of inpatients with CLC were analyzed between January 2016 and December 2019 (clinical trial registration number: ChiCTR-DDD-17013845).

The inclusion criteria were: (i) 18–80 years of age; (ii) patients with an irregular and nodular liver, along with impaired liver synthetic function, who were diagnosed with cirrhosis using imaging (ultrasonography, computed tomography, or magnetic resonance imaging) [12]; (iii) the absence of previous decompensated complications of cirrhosis including ascites, hepatic encephalopathy, or gastroesophageal variceal hemorrhage; and (iv) patients that had their data record updated (EGD, imaging, and laboratory examinations) within 3 months of this study.

Exclusion criteria were: (i) decompensated cirrhosis; (ii) primary prevention using non-selective β-blockers or endoscopic ligation; (iii) portal vein thrombosis; (iv) non-cirrhotic portal hypertension; (v) splenectomy or congenital absence of the spleen; (vi) transjugular intrahepatic portosystemic shunt procedure performed on the patient; (vii) hepatocellular carcinoma; (viii) liver transplantation; and (ix) incomplete clinical data.

### Patient evaluation

#### EGD

EGD was performed by two experienced operators using the Olympus CV-260SL processor (Olympus medical imaging, Osaka, Japan). The varices were described according to the expert guidelines [5, 13], and HRV was defined as gastroesophageal varices with a diameter ≥ 5 mm or were red-sign positive, which required non-selective beta-blockers (NSBB), ligation, or sclerotherapy to prevent bleeding.

#### Transient elastography

The procedure of LS measurement was performed using Fibroscan 520 (Echosens, Paris, France) by two senior trained professionals. The patients were in a fasting state on the morning of assessment and were placed in a supine position with the right arm in maximum abduction to make the right lobe of the liver accessible during operation. Ten measurements were obtained with a success rate of 60% and an interquartile range of less than 30%.

#### Spleen size

Patients routinely underwent abdominal ultrasound examinations to measure spleen size. The spleen diameter and thickness (measured across from the spleen hilum) were reported and recorded to calculate spleen area (SA = diameter × thickness) according to the published guidelines [14]. The patients fasted overnight. Ultrasound was then performed by professional operators who were blinded from the clinical details of the patients.

### Statistical analysis

Statistical analyses were performed using SPSS 20.0 package (IBM SPSS, Chicago, IL). Continuous data were represented by the median (interquartile range), and categorical variables by proportions. Chi-square tests or Mann–Whitney *U* tests were used for group comparisons of categorical and continuous variables. The entire cohort was divided into a training cohort (TC) (*n* = 583) and a validation cohort (VC) (*n* = 605) by the split-sample method. Variables found to be significant in univariate analyses (*p* < 0.05) were included in multivariable logistic regressions (forward selection approach for stepwise regressions) in the TC. The predicting performance of non-invasive indicators for HRV was assessed by receiver operating curve (ROC) analyses. Sensitivity (SE), specificity (SP), and negative predictive value (NPV) were calculated for each criterion under different age stratifications.

## Results

### General characteristics

**Fig. 1:**
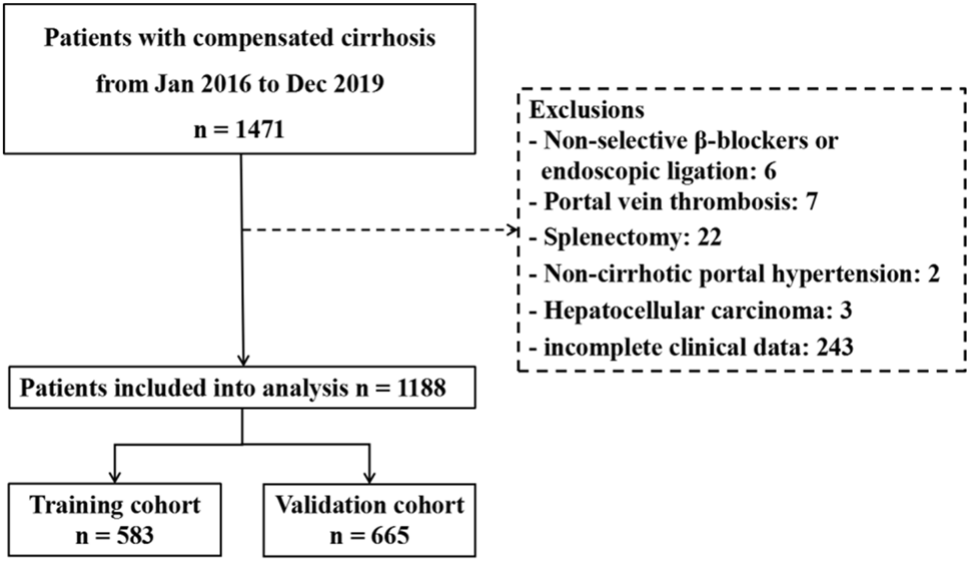
Flow chart of the study population

A total of 1,188 participants were included in the study and were divided into 583 and 605 participants in TC and VC groups, respectively. The flow chart for participant inclusion/exclusion is provided in Fig. 1. The clinical characteristics of patients in the TC and VC are listed in Table 1. The median age of patients in the entire cohort was 52 (25th to 75th percentiles, 42–59), among whom 701 (59.0%) were male, 1068 (89.9%) were Child–Pugh A class, 576 (48.5%) had varices, and 146 (12.3%) had HRV. The most common cause of cirrhosis was hepatitis B viral infection, which was found in 415 of the 583 patients (71.2%) in the TC and 446 of the 605 participants (73.7%) in the VC. The VC had similar baseline characteristics to the TC. There was no statistical difference between the TC and VC. A comparison of patients with and without HRV in the entire cohort is provided in Supporting Table S1.

**Table 1:** General characteristics and comparison of TC and VC

Variable	All (*n* = 1188)	TC (*n* = 583)	VC (*n* = 605)	*p* value
Age, years	52 (42, 59)	52 (42, 59)	52 (41, 60)	0.791
Man, *n* (%)	701 (59.0)	355 (60.9)	346 (57.2)	0.195
BMI, kg/m^2^	24.9 (22.8, 27.3)	25.0 (22.9, 27.4)	24.8 (22.7, 27.3)	0.25
Etiology, *n* (%)	–	–	–	0.552
HBV	861 (72.5)	415 (71.2)	446 (73.7)	–
Anti-varial	806 (93.6)	390 (94.0)	416 (93.3)	–
HCV	169 (14.2)	89 (15.3)	80 (13.2)	–
Anti-varial	68 (40.2)	38 (42.7)	30 (37.5)	–
Non-viral infectious	158 (13.3)	79 (13.5)	79 (13.1)	–
C-P A, *n* (%)	1068 (89.9)	530 (90.9)	538 (88.9)	0.257
ALT, U/L	49 (26, 118)	48 (26, 113)	49 (25, 125)	0.742
AST, U/L	45 (26, 102)	44 (26, 97)	47 (26, 111)	0.292
ALB, g/L	43.1 (39.0, 46.2)	43.5 (39.4, 46.1)	42.8 (38.8, 46.4)	0.189
TBIL, μmol/L	17.4 (13.1, 24.6)	17.4 (13.2, 23.9)	17.3 (13.1, 25.7)	0.753
INR	1.05 (1.00, 1.14)	1.05 (1.00, 1.13)	1.05 (1.00, 1.15)	0.666
PLT, × 10^9^/L	135 (98, 174)	135 (101, 174)	135 (98, 172)	0.514
LS, kPa	14.3 (8.8, 24.5)	14.0 (8.8, 25.7)	15.0 (9.0, 23.6)	0.369
SA, cm^2^	42.94 (34.65, 56.25)	42.56 (33.95, 56.25)	43.32 (35.12, 55.90)	0.46
Varices, *n* (%)	576 (48.5)	282 (48.4)	294 (48.6)	0.938
HRV, *n* (%)	146 (12.3)	69 (11.8)	77 (12.7)	0.64

*TC* training cohort, *VC* validating cohort, *HRV* high-risk varices, *BMI* Body mass index, *HBV* hepatitis B virus, *HCV* hepatitis C virus, *C-P* Child–Pugh class, *ALT* alanine aminotransferase, *AST* aspartate aminotransferase, *ALB* albumin, *TBIL* total bilirubin, *INR* international normalized ratio, *PLT* platelet count, *LS* liver stiffness, *SA* spleen area

### Predictors of HRV in the TC

**Fig. 2:**
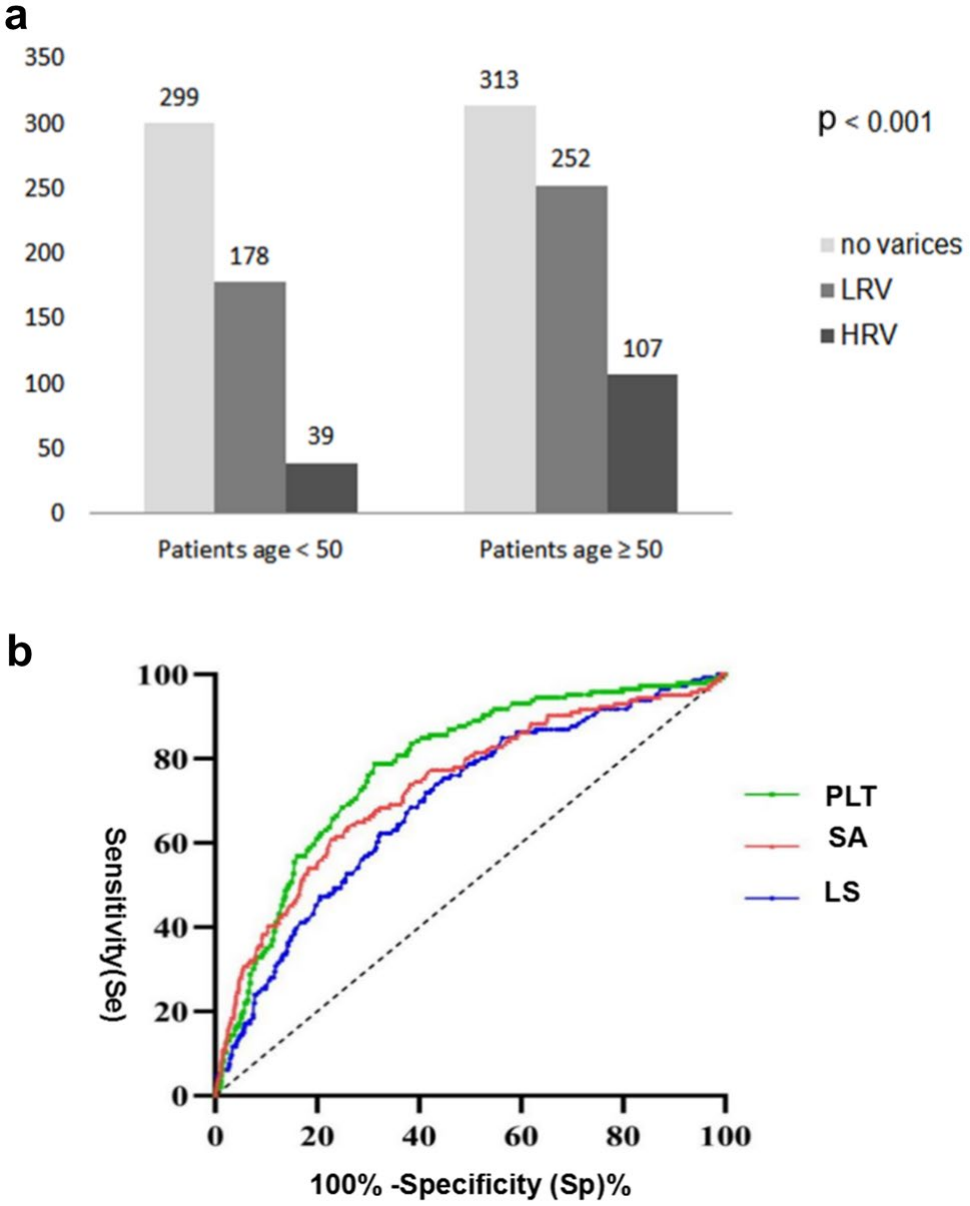
Age, PLT, SA and LS for predicting HRV. *LRV* low-risk varices, *HRV* high-risk varices, *PLT* platelet count (× 10^9^/L), *LS* liver stiffness (kPa), *SA* spleen area. **a** Prevalence of varices and high-risk varices in age-related subgroups of the entire cohort. The risk of HRV in patients ≥ 50 years was 2.387 times higher than that in patients < 50 years. **b** Efficacy of PLT, LSM and spleen area in predicting HRV. There was no statistical difference between PLT, SA and LS. Thus, SA could replace LS for build new criteria for predicting HRV

Univariate analyses were performed to detect variables associated with the presence of HRV. Table 2 shows that age, albumin, total bilirubin, international normalized ratio, PLT, LS, and SA were significantly different (all *p* < 0.05). ROC analyses showed that the area under the receiver operating characteristic (AUROC) of age was 0.605 (95% confidence interval [CI]: 0.564–0.645), and the best cut-off value was 52 years of age. To create an “easy-to-use” algorithm, 50 years of age was selected for risk stratification. Age was evaluated as dichotomous data in the multivariate analyses. The risk of HRV in patients over 50 years was 2.387-fold greater than those under 50 years of age (Fig. 2a).

**Table 2:** Predictors of HRV in TC by Univariate analysis and Multivariate analysis respectively

Variable	Univariate analysis			Multivariate analysis	
	HRV + (*n* = 69)	HRV − (*n* = 514)	*p* value	OR, 95% CI	*p* value
Age (years)	55 (48, 62)	51 (41, 58)	0.004	–	–
Age ≥ 50, *n* (%)	49 (71.0)	287 (55.8)	0.017	2.387, 1.282–4.443	0.006
Man, *n* (%)	43 (62.3)	312 (60.7)	0.796	–	–
BMI, kg/m^2^	24.9 (22.9, 27.6)	25.0 (22.9, 27.3)	0.964	–	–
C-P A, *n* (%)	63 (91.3)	467 (90.9)	0.903	–	–
ALT, U/L	42 (27, 77)	49 (26, 119)	0.258	–	–
AST, U/L	52 (28, 92)	44 (26, 99)	0.515	–	–
ALB, g/L	40.7 (37.4, 45.4)	43.7 (39.8, 46.3)	0.002	–	–
TBIL, μmol/L	18.9 (15.3, 27.1)	17.1 (12.9, 23.6)	0.017	–	–
INR	1.11 (1.05, 1.27)	1.04 (0.99, 1.12)	< 0.001	–	–
PLT, × 10^9^/L	85 (65, 116)	141 (108, 179)	< 0.001	0.986, 0.979–0.993	0.000
LS, kPa	27.0 (16.9, 36.8)	13.1 (8.3, 21.6)	< 0.001	1.034, 1.014–1.053	0.001
SA, cm^2^	59.04 (47.18,72.8)	41.19 (32.99, 53.75)	< 0.001	1.018, 1.003–1.033	0.017

*HRV* high-risk varices, *TC* training cohort, *OR* odds ratio, *CI* confidence interval, *BMI* body mass index, *C-P* Child–Pugh class, *ALT* alanine aminotransferase, *AST* aspartate aminotransferase, *ALB* albumin, *TBIL* total bilirubin, *INR* international normalized ratio, *PLT* platelet count, *LS* liver stiffness, *SA* spleen area

Multivariate analyses showed that age ≥ 50 years, LS, PLT, and SA were independently associated with HRV (Supporting Table S4). In the TC, the predicting performance of PLT, SA and LS for HRV was assessed by a receiver operating curve (ROC) analysis. The AUROC of PLT, SA and LS were 0.781 ([CI] 0.745–0.814), 0.731 ([CI] 0.694–0.767), and 0.735 ([CI] 0.697–0.770), respectively. The Delong test showed that there was no statistical difference between PLT, SA, and LS (Fig. 2b). Therefore, SA could replace LS to help establish a new criterion to predict HRV.

### Modified B6C based on age stratification

Table 3 shows the performance of the exploratory data and the new age-based criteria by adjusting the cut-off value of LS and PLT in each age-related subgroup. In patients < 50 years of age, the criteria of PLT > 100 × 10^9^/L and LS < 30 kPa maximized the number of potential EGDs that were avoided while keeping the risk of missing a HRV below the 5% threshold. For patients aged ≥ 50 years, the same result was obtained with a combination of PLT > 125 × 10^9^/L and LS < 20 kPa. We proposed to name this new algorithm the modified B6C (MB6C).

**Table 3:** Performance of the exploratory data and MB6C and AB6C classification rules based on age in the TC

Variable		Patients age < 50 (*n* = 247)			Patients age ≥ 50 (*n* = 336)	
		EGD spared, *n*, (%)	HRV missed, *n*, (%)		EGD spared, *n*, (%)	HRV missed, *n*, (%)
PLT	> 115	171 (69.2)	3 (1.8)	> 125	179 (53.3)	12 (6.7)
	> 110	175 (70.9)	3 (1.7)	> 120	192 (57.1)	13 (6.8)
	> 100	193 (78.1)	3 (1.6)	> 115	207 (61.6)	14 (6.8)
LS	< 30	204 (82.6)	9 (4.4)	< 25	251 (74.7)	23 (9.2)
	< 25	182 (73.7)	5 (2.7)	< 20	217 (64.6)	18 (8.3)
	< 20	164 (66.4)	4 (2.4)	–	–	–
SA	< 55	172 (69.6)	6 (3.5)	< 55	249 (74.1)	21 (8.4)
	< 50	155 (62.8)	3 (1.9)	< 50	222 (66.1)	17 (7.7)
	< 44	125 (50.6)	2 (1.6)	< 44	178 (53.0)	11 (6.2)
MB6C	PLT > 100 + LS < 30	171 (69.2)	1 (0.6)	PLT > 125 + LS < 20	145 (43.2)	6 (4.1)
AB6C	PLT > 100 + SA < 55	158 (64.0)	3 (1.9)	PLT > 125 + SA < 44	129 (38.4)	6 (4.7)

*MB6C* modified Baveno VI criteria, *AB6C* alternative Baveno VI criteria, *HRV* high-risk varices, *TC* training cohort, *EGD* esophagogastroduodenoscopy, *PLT* platelet count (× 10^9^/L), *LS* liver stiffness (kPa), *SA* spleen area (cm.^2^)

Table 4 reports the performance of MB6C in the VC group. In patients < 50 years of age (*n* = 269), 187 (69.5%) met the MB6C, among whom 3 (1.6%) had HRV. In patients aged ≥ 50, 123 (36.6%) met the MB6C and 4 (3.3%) had HRV. Overall, MB6C helped avoid EGD in 310 (51.2%) patients, whereas 7 (2.3%) cases of HRV were missed.

**Table 4:** Performance of the MB6C and AB6C in the VC

	VC (*n* = 605)		Patients age < 50 (*n* = 269)		Patients age ≥ 50 (*n* = 336)	
	EGD spared, *n*, (%)	HRV missed, *n*, (%)	EGD spared, *n*, (%)	HRV missed, *n*, (%)	EGD spared, *n*, (%)	HRV missed, *n*, (%)
MB6C	310 (51.2)	7 (2.3)	187 (69.5)	3 (1.6)	123 (36.6)	4 (3.3)
AB6C	297 (49.1)	8 (2.7)	174 (64.7)	2 (1.1)	123 (36.6)	6 (4.9)

*MB6C* modified Baveno VI criteria, *AB6C* alternative Baveno VI criteria, *HRV* high-risk varices, *VC* validating cohort, *EGD* esophagogastroduodenoscopy

### Construction of the alternative B6C based on age stratification

SA was selected to replace LS for building the alternative B6C (AB6C). In patients < 50 years of age, the criteria of PLT > 100 × 10^9^/L and SA < 55 cm^2^ maximized the number of potential EGDs avoided while keeping the risk of HRV missed below 5%. In patients aged ≥ 50, the AB6C used a combination of PLT > 125 × 10^9^/L and SA < 44 cm^2^ (Table 3).

The performance of AB6C in the VC is shown in Table 4. A total of 174 (67.4%) cases met the AB6C for the subgroup age < 50 (*n* = 269), among them 2 (1.1%) had HRV. The AB6C for the subgroup aged ≥ 50 was met by 123 (36.6%) cases, and among them 6 (4.9%) had HRV. Overall, the AB6C avoided 297 (49.1%) EGDs with a total of 8 (2.7%) cases of HRV that were missed.

### Validation and comparisons of B6C, EB6C, MB6C, and AB6C

In the entire cohort (*n* = 1188), a total of 377 (31.7%) patients met the B6C. Of these 377 patients, 245 (64.9%) had no varices, 126 (33.4%) were low-risk varices (LRV) (diameter ≤ 5 mm), and only 6 (1.6%) had HRV. Among the 651 (54.8%) patients that met the EB6C, 402 (61.8%) did not have varices, 225 (34.6%) had LRV, and 24 (3.7%) had HRV (Supporting Table S2).

Next, we evaluated the performance of these criteria across the entire cohort and age-related subgroups (Supporting Table S2 and Supporting Table S3). In patients < 50 years of age (*n* = 516), the B6C, EB6C, and MB6C safely helped avoid 36.8%, 59.1%, and 69.4% EGDs, respectively. In patients ≥ 50 years of age (*n* = 672), B6C safely avoided 27.8% of EGDs with a < 5% risk of missing HRV. Although the EB6C avoided more EGDs (51.5%), 6.4% of HRV were missed. The MB6C avoided more EGDs (39.9%) than B6C (27.8%) and kept the low risk of missing HRV (3.7%). Overall, among the entire cohort, MB6C helped avoid 52.7% of potential EGDs, with only 2.2% HRV that were missed.

**Fig. 3:**
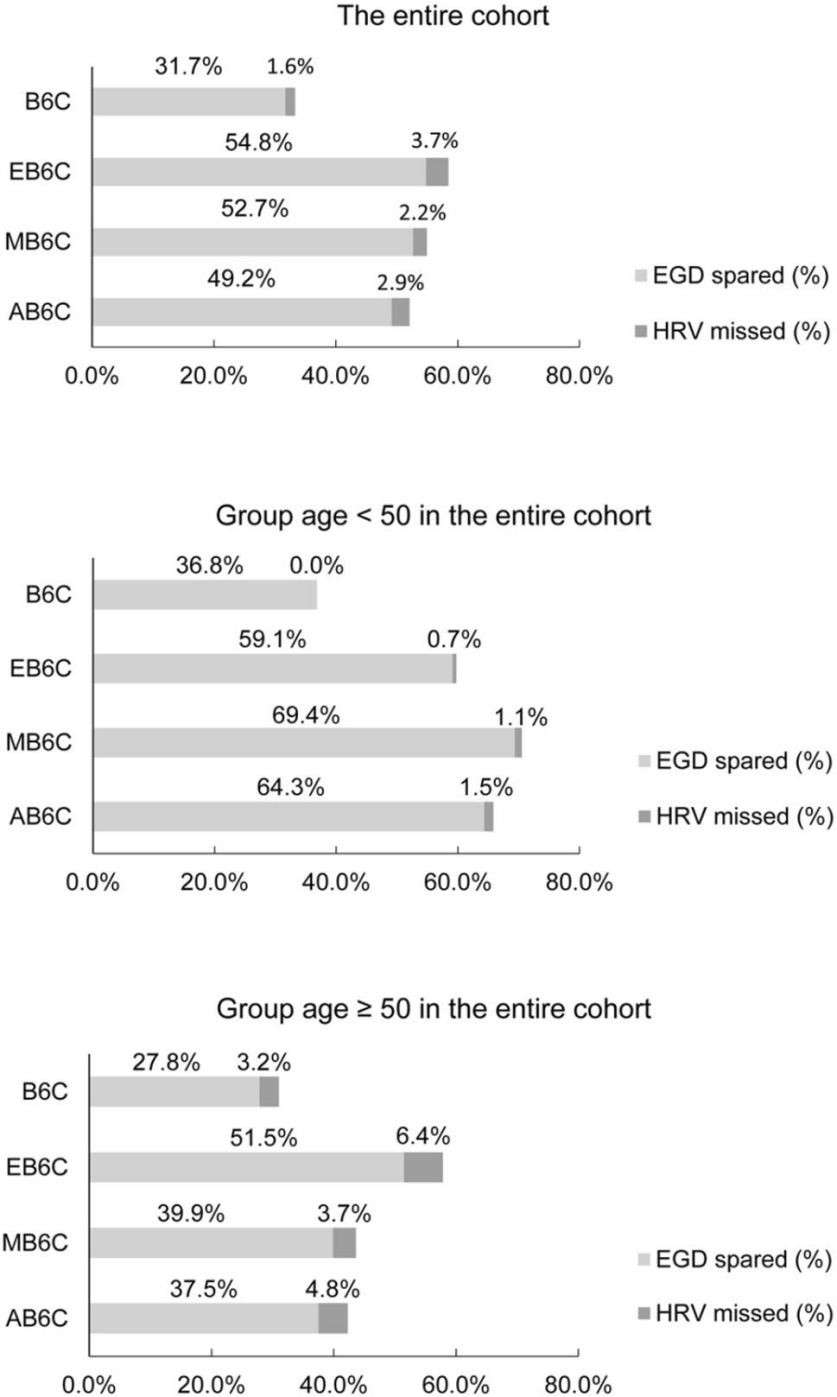
Comparison of the performance of B6C, EB6C, MB6C, and AB6C in ruling out of HRV in the entire cohort and age-related subgroups. *B6C* Baveno VI criteria, *EB6C* expanded Baveno VI criteria, *MB6C* modified Baveno VI criteria, *AB6C* alternative Baveno VI criteria, *EGD* esophagogastroduodenoscopy, *HRV* high-risk varices

AB6C also showed a good performance across the entire cohort. Using AB6C in patients aged < 50 (*n* = 516) and ≥ 50 (*n* = 672), 64.3% and 37.5% of the patients avoided EGD with 1.5% and 4.8% of HRV being missed, respectively. Overall, AB6C safely spared 49.2% EGDs while missing only 2.9% HRV (Fig. 3).

## Discussion

In this study, we proposed and validated a new screening criterion of HRV based on age stratification in patients with CLC. In similarity to Berger et al. [15], we found that the prevalence of HRV was 12.3% across the entire cohort. However, risk of HRV increased significantly with age (15.9% in patients ≥ 50 years of age versus 7.6% in patients < 50 years of age). Hepatitis B (HBV), followed by hepatitis C (HCV), were the main causes of liver cirrhosis in our cohort (86.7%), and their natural history was closely related to age. Thabut et al. [16] analyzed 891 patients with HBV and/or HCV in their prospective cohort from 35 centers in France, and found that age, an absence of viral suppression, and an unfavorable B6C status were independently associated with poorer overall survival.

It is reasonable and necessary to predict the prognosis of patients with cirrhosis by age stratification. Therefore, we hypothesized that age could be a risk stratification indicator for HRV in patients with CLC. We proposed and validated the established screening strategies based on age stratification. We first validated B6C and EB6C across the entire cohort and the age-stratified subgroups (Supporting Table S2). The number of EGDs avoided using the B6C was relatively low, and 57% of patients were misclassified. Using EB6C enhanced the number of spared EGDs among the entire cohort, but the risk of missing HRV increased to 6.4% in patients ≥ 50 years of age. Therefore, EB6C had less safety in older patients with greater risk of HRV.

Regardless of age, screening criteria for MB6C were milder than B6C. Consistent with our expectations, the screening criteria for MB6C in patients aged < 50 were milder than in patients ≥ 50 years of age. Augustin et al. [9] found that PLT > 100 × 10^9^/L and LS < 30 kPa increased the risk of missing HRV to 8.7%, but we found that this broader criterion worked well in patients < 50 years old, and sharply increased the avoidance of EGD (69.4% versus 36.8% in B6C), whereas the risk of missing HRV slightly increased (1.1%). For patients ≥ 50 years old, the more stringent screening strategy of PLT > 125 × 10^9^/L and LS < 20 kPa were adopted to keep the risk of missing HRV < 5% while the number of avoided EGDs increased by 12.1%, compared to the B6C (Supporting Tables S2 and S3).

The limited implementation of TE in many institutions has restricted the clinical use of B6C. We provided a screening strategy that does not require a LS score, offering doctors in primary hospitals a simpler method to assess and screen HRV. Ultrasound is the most commonly used liver imaging technique for its non-invasive, inexpensive, real-time imaging capability, and repeatability. SA was proposed and classified by Ishibashi et al. [17] in 1991. Their study showed that spleen index/SA correlated with resected spleen volume and guidelines highlight that a SA > 45 cm^2^ is an indicator of portal hypertension [14]. In our study, we established an alternative B6C named “AB6C” based on a combination of PLT and SA, of which the latter acted as a promising tool independently associated with the presence of HRV. Considering the wide availability of ultrasound in most medical institutions, SA could serve as an appropriate alternative to LS. AB6C retained the PLT cut-off values of MB6C, and 55 cm^2^ and 44 cm^2^ were selected as the SA cut-off values for patients aged < 50 and ≥ 50 years, respectively. AB6C achieved a similar efficacy to MB6C in ruling out HRV (Supporting Tables S2 and S3). In total, AB6C safely avoided 49.2% EGD in the entire cohort (*n* = 1188) regardless of age.

The MB6C and AB6C were formulated for patients in different age stratifications, were internally verified, and showed good performance across the entire cohort. Using these strategies may help improve safety in patients with CLC. Overall, the performance of MB6C was slightly better than AB6C, whereas both performed better than B6C and were safer than EB6C.

Our study had a few limitations. First, this was a retrospective study with data obtained from medical records, which meant that these new criteria require external verification. Second, the examinations were performed by independent operators, although they all had professional training. However, this is a common situation in clinical research.

In summary, our study demonstrated that Baveno VI criteria for avoidance of EGD in patients with CLC could be safely optimized, with LS replaced by SA. Through application of age-based MB6C and AB6C, patient avoidance of EGD achieved over 60% and close to 40% in patients aged < 50 and ≥ 50, respectively. In the entirety of the cohort, MB6C and AB6C safely avoided approximately 50% EGD, which out-performed B6C and EB6C.

## Supplementary Information

Below is the link to the electronic supplementary material.

Supplementary file1 (DOCX 110 kb)
