# Supplementary material for: Modified and alternative Baveno VI criteria based on age for ruling out high-risk varices in patients with compensated cirrhosis
Source: Hepatol Int. 2022 Jun 21;16(4):936–43. doi: 10.1007/s12072-022-10359-y (PMC9349062; doi:10.1007/s12072-022-10359-y)
Supplement: Supplementary file 1 — Supplementary file1 (DOCX 110 kb) [file 12072_2022_10359_MOESM1_ESM.docx]

**Supplementary materials**

|  | Global  (n=1188) | HRV+  (n=146) | HRV-  (n=1042) | *P* |
| --- | --- | --- | --- | --- |
| Age | 52 (42,59) | 56 (48,63) | 51 (41,59) | < 0.001 |
| Age ≥ 50, n (%) | 672 (56.6 ) | 107 (73.2 ) | 565 (54.2 ) | < 0.001 |
| Male, n (%) | 701 (59.0 ) | 88 (60.3 ) | 613 (58.8 ) | 0.74 |
| BMI, Kg/m^2^ | 24.9 (22.8,27.3) | 24.7 (22.9,27.3) | 25.0 (22.8,27.3) | 0.68 |
| Etiology, n (%) | - | - | - | 0.117 |
| HBV | 861 (72.5) | 97 (66.4) | 764 (73.3) | - |
| anti-varial | 806,(93.6) | 92(94.8) | 714(93.5) |  |
| HCV | 169 (14.2) | 22 (15.1) | 147 (14.1) | - |
| anti-varial | 68,(40.2) | 13(59.1) | 55(37.4) |  |
| Non-viral | 158 (13.3) | 27 (18.5) | 131 (12.6) | - |
| infectious |  |  |  |  |
| C-P A, n (%) | 1068 (89.9) | 128 (87.7) | 940 (90.2) | 0.34 |
| ALT, U/L | 49 (26,118) | 42 (24,89) | 50 (26,125) | 0.066 |
| AST, U/L | 45 (26,102) | 52 (30,101) | 45 (26,102) | 0.252 |
| ALB, g/L | 43.1(39.0,46.2) | 40.0(35.8,43.7) | 43.4 (39.5,46.5) | < 0.001 |
| TBIL, μmol/L | 17.4(13.1,24.6) | 21.4(15.5,31.5) | 17.0(12.9,23.6) | < 0.001 |
| INR | 1.05(1.00,1.14) | 1.14(1.04,1.27) | 1.04(1.00,1.12) | < 0.001 |
| PLT, ×10^9^/L | 135(98,174) | 88(67,112) | 141(106,179) | < 0.001 |
| LS, kPa | 14.3(8.8,24.5) | 22.3(14.9,34.9) | 13.6(8.6,22.1) | < 0.001 |
| SA, cm^2^ | 42.94(34.65,56.25) | 58.90(45.11,75.12) | 41.91(33.60,53.72) | < 0.001 |
| Varices,n (%) | 576 (48.5) | 146 (100.0) | 430 (41.3 ) | - |

**Table S1** Comparison of HRV and Non-HRV in the entire cohort

HRV: High-risk varices; BMI: Body mass index; HBV: Hepatitis B virus; HCV: Hepatitis C virus; C-P: Child-Pugh class; ALT: Alanine aminotransferase; AST: Aspartate aminotransferase; ALB: Albumin; TBIL: Total bilirubin; INR: International normalized ratio; PLT: Platelet count; LS: Liver stiffness; SA: Spleen area

**Table S2** Performance of each criteria in ruling out HRV in the Group Age < 50 (n = 247) of TC

|  |  | EGD spared  (%) | HRV missed (%) | Se | Sp | Accuracy | PPV | NPV | LR+ | LR- |
| --- | --- | --- | --- | --- | --- | --- | --- | --- | --- | --- |
| PLT | > 115 | 171 (69.2) | 3 (1.8) | 0.85 | 0.74 | 0.75 | 0.22 | 0.98 | 3.27 | 0.20 |
|  | > 110 | 175 (70.9) | 3 (1.7) | 0.85 | 0.76 | 0.77 | 0.24 | 0.98 | 3.51 | 0.20 |
|  | > 100 | 193 (78.1) | 3 (1.6) | 0.85 | 0.84 | 0.84 | 0.31 | 0.98 | 5.21 | 0.18 |
| LS | < 30 | 204 (82.6) | 9 (4.4) | 0.55 | 0.86 | 0.83 | 0.26 | 0.96 | 3.90 | 0.52 |
|  | < 25 | 182 (73.7) | 5 (2.7) | 0.75 | 0.78 | 0.78 | 0.23 | 0.97 | 3.41 | 0.32 |
|  | < 20 | 164(66.4) | 4 (2.4) | 0.80 | 0.70 | 0.71 | 0.19 | 0.98 | 2.71 | 0.28 |
| SA | < 55 | 172 (69.6) | 6 (3.5) | 0.70 | 0.73 | 0.73 | 0.19 | 0.97 | 2.60 | 0.41 |
|  | < 50 | 155 (62.8) | 3 (1.9) | 0.85 | 0.67 | 0.68 | 0.18 | 0.98 | 2.57 | 0.22 |
|  | < 44 | 125 (50.6) | 2 (1.6) | 0.90 | 0.54 | 0.57 | 0.15 | 0.98 | 1.96 | 0.18 |
| MB6C | PLT > 100 + LS < 30 | 171 (69.2) | 1 (0.6) | 0.95 | 0.75 | 0.77 | 0.25 | 0.99 | 3.78 | 0.07 |
| AB6C | PLT > 100 + SA <55 | 158 (64.0) | 3 (1.9) | 0.85 | 0.68 | 0.70 | 0.19 | 0.98 | 2.68 | 0.22 |
| B6C | PLT > 150 + LS < 20 | 90 (36.4) | 0 (0.0) | 1.00 | 0.40 | 0.45 | 0.13 | 1.00 | 1.66 | 0.00 |
| EB6C | PLT > 110 + LS < 25 | 146 (59.1) | 0 (0.0) | 1.00 | 0.64 | 0.67 | 0.20 | 1.00 | 2.80 | 0.00 |

HRV: High-risk varices; TC: Trianing cohort; EGD: Esophagogastroduodenoscopy; Se: Sensitivity; Sp: Specificity; PPV: Positive predictive value; NPV: Negative predictive value; LR+: Positive likelihood ratio; LR−: Negative likelihood ratio; PLT: Platelet count; LS: Liver stiffness; SA: Spleen area; MB6C: Modified Baveno VI criteria; AB6C: Alternative Baveno VI criteria; B6C: Baveno VI criteria; EB6C: Expanded Baveno VI criteria;

**Table S3** Performance of each criteria in ruling out HRV in the Group Age ≥50 (n=336) of TC

|  |  | EGD  spared (%) | HRV missed (%) | Se | Sp | Accuracy | PPV | NPV | LR+ | LR- |
| --- | --- | --- | --- | --- | --- | --- | --- | --- | --- | --- |
| PLT | > 125 | 179(53.3) | 12(6.7) | 0.76 | 0.58 | 0.61 | 0.24 | 0.93 | 1.81 | 0.42 |
|  | > 120 | 192 (57.1) | 13(6.8) | 0.73 | 0.62 | 0.64 | 0.25 | 0.93 | 1.95 | 0.43 |
|  | > 115 | 207(61.6) | 14(6.8) | 0.71 | 0.67 | 0.68 | 0.27 | 0.93 | 2.18 | 0.42 |
| LS | < 25 | 251(74.7) | 23(9.2) | 0.53 | 0.79 | 0.76 | 0.31 | 0.91 | 2.58 | 0.59 |
|  | < 20 | 217 (64.6) | 18(8.3) | 0.63 | 0.69 | 0.68 | 0.26 | 0.92 | 2.06 | 0.53 |
| SA | < 55 | 249(74.1) | 21(8.4) | 0.57 | 0.79 | 0.76 | 0.32 | 0.92 | 2.78 | 0.54 |
|  | < 50 | 222(66.1) | 17(7.7) | 0.65 | 0.71 | 0.71 | 0.28 | 0.92 | 2.29 | 0.49 |
|  | < 44 | 178(53.0) | 11(6.2) | 0.78 | 0.58 | 0.61 | 0.24 | 0.94 | 1.85 | 0.39 |
| MB6C | PLT > 125 + LS < 20 | 45(43.2) | 6(4.1) | 0.88 | 0.48 | 0.54 | 0.23 | 0.96 | 1.70 | 0.25 |
| AB6C | PLT > 125 + SA <44 | 129(38.4) | 6(4.7) | 0.88 | 0.43 | 0.49 | 0.21 | 0.95 | 1.54 | 0.29 |
| B6C | PLT > 150 + LS < 20 | 107(31.8) | 4(3.7) | 0.92 | 0.36 | 0.44 | 0.20 | 0.96 | 1.43 | 0.23 |
| EB6C | PLT > 110 + LS < 25 | 183(54.5) | 9(4.9) | 0.82 | 0.61 | 0.64 | 0.26 | 0.95 | 2.07 | 0.30 |

HRV: High-risk varices; TC: Trianing cohort; EGD: Esophagogastroduodenoscopy; Se: Sensitivity; Sp: Specificity; PPV: Positive predictive value; NPV: Negative predictive value; LR+: Positive likelihood ratio; LR−: Negative likelihood ratio; PLT: Platelet count; LS: Liver stiffness; SA: Spleen area; MB6C: Modified Baveno VI criteria; AB6C: Alternative Baveno VI criteria; B6C: Baveno VI criteria; EB6C: Expanded Baveno VI criteria;

**Table S4** Performance of each criteria in ruling out HRV in the VC and age-related subgroup

|  |  | EGD  spared (%) | HRV missed (%) | Se | Sp | Accuracy | PPV | NPV | LR+ | LR- |
| --- | --- | --- | --- | --- | --- | --- | --- | --- | --- | --- |
| All VC | MB6C | 310 (51.2) | 7 (2.3) | 0.91 | 0.57 | 0.62 | 0.24 | 0.98 | 2.13 | 0.16 |
| ( n = 605 ) | AB6C | 297 (49.1) | 8 (2.7) | 0.90 | 0.55 | 0.59 | 0.22 | 0.97 | 1.98 | 0.19 |
|  | B6C | 180 (29.8) | 2 (1.1) | 0.97 | 0.34 | 0.42 | 0.18 | 0.99 | 1.47 | 0.08 |
|  | EB6C | 322 (53.2) | 15 (4.7) | 0.81 | 0.58 | 0.61 | 0.22 | 0.95 | 1.92 | 0.34 |
| Age <50 | MB6C | 187 (69.5) | 3 (1.6) | 0.84 | 0.74 | 0.74 | 0.20 | 0.98 | 3.19 | 0.21 |
| ( n = 269 ) | AB6C | 174 (64.7) | 2 (1.1) | 0.89 | 0.69 | 0.70 | 0.18 | 0.99 | 2.87 | 0.15 |
|  | B6C | 100 (37.2) | 0 (0.0) | 1.00 | 0.40 | 0.44 | 0.11 | 1.00 | 1.67 | 0.00 |
|  | EB6C | 159 (59.1) | 2 (1.3) | 0.89 | 0.63 | 0.65 | 0.15 | 0.99 | 2.41 | 0.17 |
| Age ≥50 | MB6C | 123 (36.6) | 4 (3.3) | 0.93 | 0.43 | 0.51 | 0.25 | 0.97 | 1.63 | 0.16 |
| ( n = 336 ) | AB6C | 123 (36.6) | 6 (4.9) | 0.90 | 0.42 | 0.50 | 0.24 | 0.95 | 1.55 | 0.25 |
|  | B6C | 80 (23.8) | 2 (2.5) | 0.97 | 0.28 | 0.40 | 0.22 | 0.98 | 1.34 | 0.12 |
|  | EB6C | 163 (48.5) | 13 (8.0) | 0.78 | 0.54 | 0.58 | 0.26 | 0.92 | 1.69 | 0.42 |

HRV: High-risk varices; VC: Validating cohort; EGD: Esophagogastroduodenoscopy; Se: Sensitivity; Sp: Specificity; PPV: Positive predictive value; NPV: Negative predictive value; LR+: Positive likelihood ratio; LR−: Negative likelihood ratio; PLT: Platelet count; LS: Liver stiffness; SA: Spleen area; MB6C: Modified Baveno VI criteria; AB6C: Alternative Baveno VI criteria; B6C: Baveno VI criteria; EB6C: Expanded Baveno VI criteria

**Table S5** Performance of B6C and EB6C in ruling out HRV in the entire cohort and age-related subgroups

|  | entire cohort (n=1188) | |  | Patients age <50  (n=516) | |  | Patients age ≥ 50  (n=672) | |
| --- | --- | --- | --- | --- | --- | --- | --- | --- |
|  | B6C | EB6C |  | B6C | EB6C |  | B6C | EB6C |
| EGD Spared,  n (%) | 377 (31.7) | 651 (54.8) |  | 190 (36.8) | 305 (59.1) |  | 187 (27.8) | 346 (51.5) |
| HRV missed,  n (%) | 6 (1.6) | 24 (3.7) |  | 0 (0.0) | 2 (0.7) |  | 6 (3.2) | 22 (6.4) |
| Se,% | 95.90 | 83.60 |  | 100.00 | 94.90 |  | 94.40 | 79.40 |
| Sp,% | 35.60 | 60.20 |  | 39.80 | 63.50 |  | 32.00 | 57.30 |
| Accuracy,% | 43.00 | 63.00 |  | 44.40 | 65.90 |  | 42.00 | 60.90 |
| PPV,% | 17.30 | 22.70 |  | 12.00 | 17.50 |  | 20.80 | 26.10 |
| NPV,% | 98.40 | 96.30 |  | 100.00 | 99.30 |  | 96.80 | 93.60 |
| LR+ | 1.49 | 2.10 |  | 1.66 | 2.60 |  | 1.39 | 1.86 |
| LR- | 0.12 | 0.27 |  | 0.00 | 0.08 |  | 0.18 | 0.36 |

B6C: Baveno VI criteria; EB6C: Expanded Baveno VI criteria; HRV: High- risk varices; EGD: Esophagogastroduodenoscopy; Se: Sensitivity; Sp: Specificity; PPV: Positive predictive value; NPV: Negative predictive value; LR+: Positive likelihood ratio; LR−: Negative likelihood ratio

**Table S6** Performance of MB6C and AB6C in ruling out HRV in the entire cohort and age-related subgroup

|  | entire cohort (n=1188) | |  | Patients age <50 (n=516) | |  | Patients age ≥ 50  (n=672) | |
| --- | --- | --- | --- | --- | --- | --- | --- | --- |
|  | MB6C | AB6C |  | MB6C | AB6C |  | MB6C | AB6C |
| EGD Spared,  n (%) | 626 (52.7) | 584 (49.2) |  | 358 (69.4 ) | 332 (64.3) |  | 268 (39.9 ) | 252 (37.5 ) |
| HRV missed,  n (%) | 14 (2.2) | 17 (2.9) |  | 4 (1.1 ) | 5 (1.5 ) |  | 10 (3.7 ) | 12 (4.8 ) |
| Se,% | 90.40 | 88.40 |  | 89.70 | 87.20 |  | 90.70 | 88.80 |
| Sp,% | 58.70 | 54.40 |  | 74.20 | 68.60 |  | 45.70 | 42.50 |
| Accuracy,% | 62.60 | 58.60 |  | 75.40 | 70.00 |  | 52.80 | 49.90 |
| PPV,% | 23.50 | 21.40 |  | 22.20 | 18.50 |  | 24.00 | 22.60 |
| NPV,% | 97.80 | 97.10 |  | 98.90 | 98.50 |  | 96.30 | 95.20 |
| LR+ | 2.19 | 1.94 |  | 3.48 | 2.77 |  | 1.67 | 1.54 |
| LR- | 0.16 | 0.21 |  | 0.14 | 0.19 |  | 0.2 | 0.26 |

MB6C: Modified Baveno VI criteria; AB6C: Alternative Baveno VI criteria; HRV: High-risk varices; EGD: Esophagogastroduodenoscopy; Se: Sensitivity; Sp: Specificity; PPV: Positive predictive value; NPV: Negative predictive value; LR+: Positive likelihood ratio; LR−: Negative likelihood ratio

**Fig S1** Prevalence of varices and high-risk varices in age-related subgroups of the entire cohort


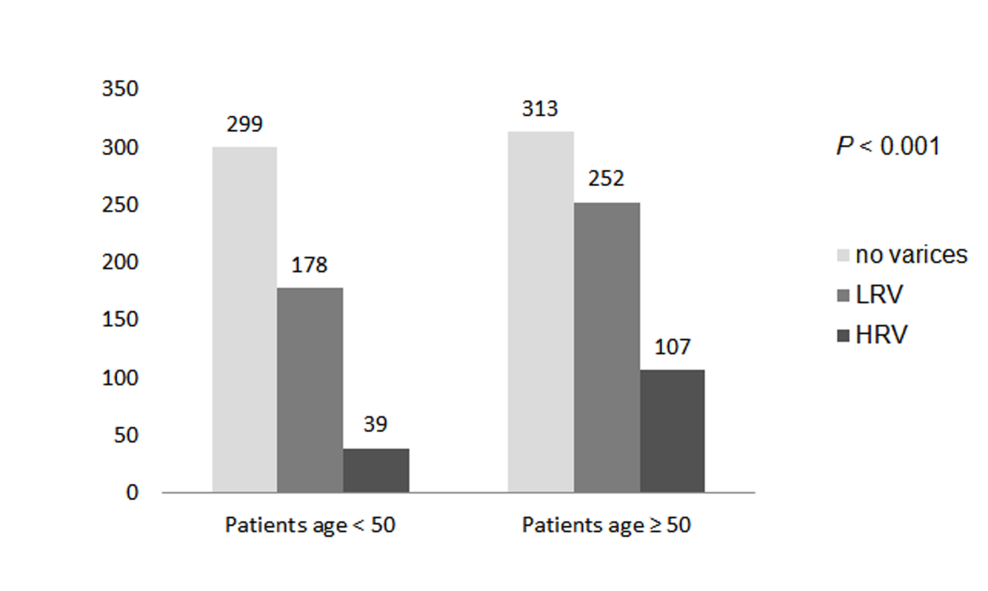


LRV: Low-risk varices; HRV: High-risk varices;
